# Male patients with TERT mutation may be more likely to benefit from immunotherapy, especially for melanoma

**DOI:** 10.18632/aging.103684

**Published:** 2020-09-10

**Authors:** Jia Li, Zhaoyan Li, Chenyue Zhang, Chenxing Zhang, Haiyong Wang

**Affiliations:** 1 Department of Integrated Chinese and Western Medicine, Affiliated Cancer Hospital of Zhengzhou University and Henan Cancer Hospital, Zhengzhou 450008, China; 2 Department of Oncology, Yueyang Hospital of Integrated Traditional Chinese and Western Medicine, Shanghai University of Traditional Chinese Medicine (TCM), Shanghai 200437, China; 3 Department of Integrated Therapy, Fudan University Shanghai Cancer Center, Shanghai Medical College, Shanghai 00032, China; 4 Department of Nephrology, Shanghai Children's Medical Center, Shanghai Jiao Tong University School of Medicine, Shanghai 200127, China; 5 Department of Internal Medicine-Oncology, Shandong Cancer Hospital and Institute, Shandong First Medical University and Shandong Academy of Medical Sciences, Jinan 250117, China

**Keywords:** sex disparity, TERT mutation, immunotherapy, melanoma

## Abstract

Genomic mutation may be key factors for sex-biased disparities in cancer diagnosis, prognosis and prediction of treatment response. Current study has revealed that sex-based dimorphism on the efficacy of immune checkpoint inhibitors (ICIs) in various cancers and confirmed that male patients can benefit more from immunotherapy. However, only a subset of male patients responds well to ICIs. Therefore, biomarkers are desperately needed to identify the group of patients who may be more likely to benefit from ICIs. With the availability of the cBioPortal database, we identified that TERT mutation may serve as a sex-specific cancer biomarker and TERT mutation frequency of melanoma was higher in male patients. Notably, we found that male patients with TERT mutation may be more likely to benefit from immunotherapy (*p* = 0.006), especially for melanoma (*p* < 0.001). Therefore, our research provides a possible direction for the exploration of immunotherapy prediction biomarkers based on sex difference.

## INTRODUCTION

Sex is defined by biological differences in the chromosomes, reproductive organs, and sex steroid levels between men and women, while gender is determined by behaviors and activity differences in human society or culture between male and female [1]. In many cancer types, significant sex disparities in cancer incidence, survival, and prevalence and treatment responses have also been reported [2, 3]. Sex disparities in cancer are presumably induced by sex hormones, especially estrogen, which are known to modulate gene expression in many cancers [4, 5]. Genomic mutation, may be key factors for sex-biased disparities in mortality and incidence of cancers [6]. Most prior research on genomic determinants of sex differences have focused on TMB [7]. However, the mutant genes related to sex disparity have yet to be elucidated.

Immune checkpoint inhibitors (ICIs) have revolutionized cancer treatment, showing higher efficacy than standard therapies in the management of several difficult-totreat malignancies, including malignant melanoma, non-small-cell lung cancer, bladder cancer, and head and neck cancer, among many others [8]. Recently, sex-based ICI efficacy dimorphism has been found in various cancers, suggesting that male cancer patients benefit from ICIs more than female cancer patients [9, 10]. However, only a subset of patients respond well to ICIs. Recent studies suggested that TMB was an efficient pan-cancer biomarker for ICI response and was preferentially useful in female patients [11]. However, due to the non-uniform calculation, various sequencing approaches and exorbitant expenses, TMB is not an optimal indicator to ICIs responses in some types of cancer. Therefore, new biomarkers are desperately needed to identify the group of patients who may be more likely to benefit from ICIs.

Telomerase reverse transcriptase (TERT) mutations lead to aberrantly upregulate TERT expression, and ultimately enable telomere maintenance, which achieve unlimited proliferative capacity of tumor cells [12, 13]. Moreover, TERT mutations are identified as biomarkers of tumor aggressiveness and poor prognosis in several human cancer types [14–17]. Recently, a study on bladder cancer suggested that TERT promoter mutation has been identified as a potential predictive marker of Bacillus Calmette-Guérin (BCG) treatment which was regarded as one of the first and most successful of all oncological immunotherapies [18]. However, a comprehensive analysis of TERT mutation and their predictive value for ICIs has never been elucidated to date.

With the availability of the cBioPortal database, we firstly compared the mutant genes related to sex disparity, and then analyzed the frequency of TERT mutation between different sex groups in different cancer types. Finally, we explored the effect of TERT mutation and sex disparity on the efficacy of immunotherapy in pan cancer, especially melanoma.

## RESULTS

### The differential mutant genes related to sex disparity

**Figure 1:**
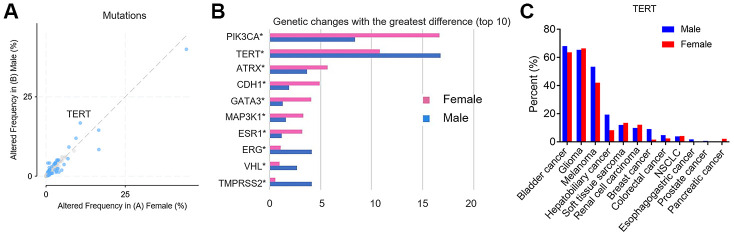
**The differential mutant genes between female vs male group.** (**A**) Scatter plots, the altered frequency of genetic mutations between female vs male group (%). The X axis represents altered frequency in female. The Y axis represents altered frequency in male. The blue dots indicate that the *p* and q values are statistically significant. (**B**) The top 10 genes with the greatest difference of mutations between female vs male group. The X axis represents genetic changes with the greatest difference. The Y axis represents differential mutant genes. The red histogram indicates female and the blue histogram indicates male. (**C**) The frequency of TERT mutations between female vs male group in different cancer types. X-axis represents common types of cancer. The Y axis represents percent of TERT mutation. The red histogram indicates female and the blue histogram indicates male.

Scatter plots all showed differences in genetic mutations between different sex groups (Figure 1A). The blue dots indicated that the *p* and q values were statistically significant, and the gray dots were not significant. We also showed the top 10 genes with the greatest difference of mutations in female and male. As shown in Figure 1B, the mutation frequency of PIK3CA, ATRX, CDH1, GATA3, MAP3K1, and ESR1 was higher in the female than in the male group (*p* <0.05). In TERT, ERG, VHL, and TMPRSS2, the mutation frequency was higher in the male than in the female group, and the difference was also statistically significant (*p* <0.05).

We further analyzed the frequency of TERT mutations between different sex groups in different cancer types. Figure 1C showed that Bladder Cancer, Glioma and Melanoma were the top three of TERT mutation frequency, while Esophagogastric cancer, Prostate cancer, and Pancreatic cancer were the last three. Notably, for Melanoma and Hepatobiliary cancer, TERT mutation frequency was higher in male patients than female patients.

### Relationship between TERT mutation and sex disparity and immunotherapy

**Figure 2:**
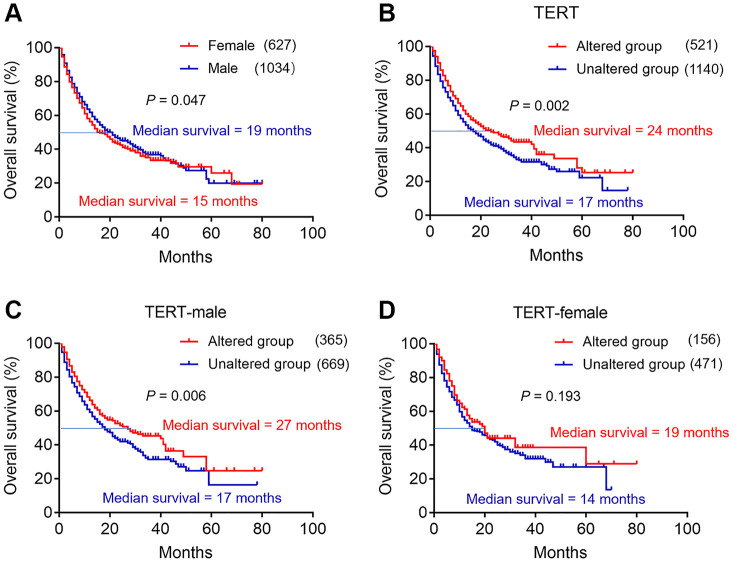
**The effect of TERT mutation and sex disparity on the efficacy of immunotherapy in cBioPortal database.** (**A**) Kaplan-Meier survival analysis between female and male patients in ICI treatment cohort. The red curve represents female group, and the blue curve represents male group. (**B**) Kaplan-Meier survival analysis between TERT altered and unaltered group in ICI treatment cohort. The red curve represents TERT altered group, and the blue curve represents unaltered group. (**C**) Kaplan-Meier survival analysis between TERT altered and unaltered group in the TERT-male cohort. The red curve represents TERT altered group, and the blue curve represents unaltered group. (**D**) Kaplan-Meier survival analysis between TERT altered and unaltered group in the TERT-female cohort. The red curve represents TERT altered group, and the blue curve represents unaltered group.

We first explored the association between sex and overall survival (OS) in the immunotherapy cohort. As shown in Figure 2A, male patients had significantly better median OS than female patients (19 months vs 15 months, *p* =0.047). From the above results, we found that TERT mutation was related to sex disparity. We also explored the predictive value of TERT mutation on the efficacy of immunotherapy. Surprisingly, patients in the TERT altered group showed a significantly longer median OS than unaltered group (Figure 2B, 24 months vs 17 months, *p* =0.002).

In order to further understand whether male and female patients with TERT mutation can benefit from immunotherapy. We next conducted subgroup analysis in the TERT-male and TERT-female cohort. Notably, in TERT-male group, male patients in the TERT altered group had longer median OS than unaltered group (Figure 2C, 27 months vs 17 months, *p* =0.006). Similar results were obtained in TERT-female cohort, female patients in the TERT altered group had longer median OS trend than unaltered group, but not statistically significant (Figure 2D, 19 months vs 14 months, *p* =0.193).

### Relationship between TERT mutation and immunotherapy in different sex groups of melanoma

**Figure 3:**
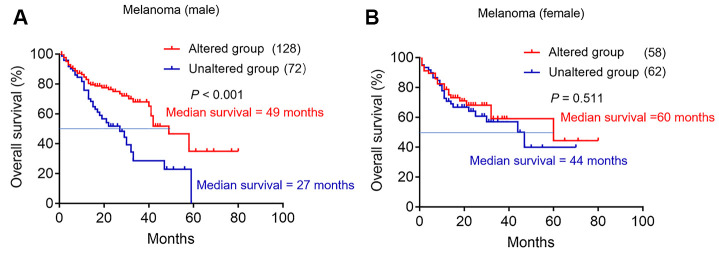
**The effect of TERT mutation on the efficacy of immunotherapy in melanoma database.** (**A**) Kaplan-Meier survival analysis between TERT altered and unaltered group in the male cohort. The red curve represents TERT altered group, and the blue curve represents unaltered group. (**B**) Kaplan-Meier survival analysis between TERT altered and unaltered group in the female cohort. The red curve represents TERT altered group, and the blue curve represents unaltered group.

To explore the predictive value of TERT mutation on the efficacy of immunotherapy in certain cancer types. The cancer types including non-small cell lung cancer, melanoma, bladder cancer, renal cell carcinoma, head and neck cancer, esophagogastric cancer, glioma and colorectal cancer were further analyzed. However, the result showed that only male melanoma patients in the TERT altered group had longer median OS than unaltered group (Figure 3A, 49 months vs 27 months, *p*<0.001). Similar to pan cancer analysis, the statistically OS significant was not found for female melanoma patients in the TERT altered group (Figure 3B, 60 months vs 44 months, *p* =0.511). Other tumor types did not get a positive result, and the data were not given.

## DISCUSSION

Sex disparities were biologically related to cancer diagnosis, prognosis, severity, and prediction of treatment response [2, 3]. Genomics studies showed a variation in the expression of autosomal genes between males and females [19, 20]. TMB, as a biomarker of response to ICIs, has been considered to be more predictive of the efficacy of immunotherapy in female patients [11]. However, due to the limitations of TMB application, we needed to explored new biomarkers related to sex disparity to better predict ICIs response.

In our study, we showed the top 10 genes with the greatest difference of mutations, which were related to sex disparity. This variation (genetic mutation) may help facilitate the identification of sex-specific cancer biomarkers. We found that PIK3CA and TERT were genes with the greatest differences between different sex groups. Strikingly, TERT mutation could be frequent in many malignancies and be identified as biomarkers of tumor aggressiveness and poor prognosis in several human cancer types [14–17]. We further analyzed the frequency of TERT mutations between different sex groups in different cancer types. The result showed that Bladder Cancer, Glioma and Melanoma were the top three of TERT mutation frequency, while Esophagogastric cancer, Prostate cancer, and Pancreatic cancer were the last three. Consistent results have also been confirmed the high prevalence of TERT mutation in bladder cancer, Glioma and Melanoma and a lower mutation frequency of breast cancer and prostate cancer, although they occurred with different frequencies [21–25]. Moreover, we also found that TERT mutation frequency of Melanoma and Hepatobiliary cancer was higher in male patients than female patients. Lee et al analyzed TERT promoter mutation in 162 tumor samples of the patients with HCC by sequencing and real-time PCR, respectively. The result showed that the TERT promoter mutation rate was 28.8% (46/160) in HCC and was associated with males (*p* =0.027) [26]. Egberts et al also found highly recurrent TERT promoter mutations in malignant melanomas, and the prevalence of its TERT-promoter mutations was associated with the patient's sex [23 (51.1%) men, 10 (21.3%) women; *p* = 0.004] [27]. The above results suggested that TERT mutation might be closely related to sex, although efforts are still needed to evaluate the clinical utility of sex-biased TERT mutation in a larger patient cohort.

But for patients who received ICIs, what was the relationship between TERT mutation and OS? We evaluated the effect of TERT mutation and sex disparity on the efficacy of immunotherapy. Our results demonstrated that male patients and patients with TERT mutation all had significantly better median OS. More importantly, male patients with TERT mutation had longer median OS. Current study has revealed that sex-based dimorphism on the efficacy of ICIs in various cancers and confirmed that male patients can benefit more from immunotherapy [8, 28, 29]. As we all know, not all male patients can benefit from immunotherapy, and new biomarkers are still needed to predict the efficacy of immunotherapy. Our study reported that male patients with TERT mutation may be more likely to benefit from immunotherapy in a multitude of cancers.

Our study has not only found a high frequency of TERT mutations in melanoma, but also a higher frequency of mutations in male melanoma patients. In addition, effect of sex on immunotherapy may exist in specific cancer types rather than all cancer types [30, 31]. TERT mutation should also be like this. Our result showed that only male melanoma patients with TERT mutation had longer median OS. Therefore, male melanoma patients with TERT mutation may benefit from immunotherapy.

This study also has certain limitations. Firstly, our study was only a bioinformatic based on public databases. The next step is to confirm whether male patients with TERT mutation could benefit from immunotherapy through prospective or retrospective studies. Secondly, the immunotherapy data we collected for each cancer type was very limited. Although we did immunotherapy analyses in certain cancer types, the same conclusion was only obtained in melanoma. As for other tumor types, we need to determine whether male patients with TERT mutations benefit from immunotherapy.

In conclusion, using the cBioPortal database, we identified that TERT mutation may serve as a sex-specific cancer biomarker and TERT mutation frequency of melanoma was higher in male patients. Notably, we found that male patients with TERT mutation may be more likely to benefit from immunotherapy, especially for melanoma. However, the potential role of TERT mutation as biomarkers for immunotherapy are still needed to confirm by further clinical trials.

## MATERIALS AND METHODS

All data in this study were selected from the cBioPortal database (https://www.cbioportal.org) [32]. To analyze the differences of gene mutation according to sex disparity, we used the MSK-IMPACT Clinical Sequencing Cohort [33], which contain 10336 patients/10945 samples with available mutation and sex status information.

For the relationship analysis between gender, TERT mutation and immunotherapy, we also selected TMB and Immunotherapy Cohort [34], which contains 1661 patients with various cancer types (including 320 cases of melanoma) with available survival, immunotherapy, TERT mutation and sex status information. Data between two groups were compared using unpaired t-test or Wilcoxon rank-sum test. Kaplan-Meier method was used to calculate the survival probability and log-rank test was used to compare the survival curves. All reported *p* values are two-tailed, and for all analyses, *p* <0.05 is considered statistically significant. This study was mainly based on the cBioPortal database and personal privacy information was not involved, so the informed consent was not needed.

## References

[1] Clayton JA, Tannenbaum C. Sex and gender reporting in research-reply. JAMA. 2017; 317:975. 10.1001/jama.2017.0151 28267852

[2] Zhu Y, Shao X, Wang X, Liu L, Liang H. Sex disparities in cancer. Cancer Lett. 2019; 466:35–38. 10.1016/j.canlet.2019.08.017 31541696

[3] Cook MB, McGlynn KA, Devesa SS, Freedman ND, Anderson WF. Sex disparities in cancer mortality and survival. Cancer Epidemiol Biomarkers Prev. 2011; 20:1629–37. 10.1158/1055-9965.EPI-11-0246 21750167 PMC3153584

[4] Özdemir BC, Dotto GP. Sex hormones and anticancer immunity. Clin Cancer Res. 2019; 25:4603–10. 10.1158/1078-0432.CCR-19-0137 30890551

[5] Kim HI, Lim H, Moon A. Sex differences in cancer: epidemiology, genetics and therapy. Biomol Ther (Seoul). 2018; 26:335–42. 10.4062/biomolther.2018.103 29949843 PMC6029678

[6] Mostertz W, Stevenson M, Acharya C, Chan I, Walters K, Lamlertthon W, Barry W, Crawford J, Nevins J, Potti A. Age- and sex-specific genomic profiles in non-small cell lung cancer. JAMA. 2010; 303:535–43. 10.1001/jama.2010.80 20145230

[7] Gupta S, Artomov M, Goggins W, Daly M, Tsao H. Gender disparity and mutation burden in metastatic melanoma. J Natl Cancer Inst. 2015; 107:djv221. 10.1093/jnci/djv221 26296643 PMC4643631

[8] Ribas A, Wolchok JD. Cancer immunotherapy using checkpoint blockade. Science. 2018; 359:1350–55. 10.1126/science.aar4060 29567705 PMC7391259

[9] Conforti F, Pala L, Bagnardi V, De Pas T, Martinetti M, Viale G, Gelber RD, Goldhirsch A. Cancer immunotherapy efficacy and patients’ sex: a systematic review and meta-analysis. Lancet Oncol. 2018; 19:737–46. 10.1016/S1470-2045(18)30261-4 29778737

[10] Wang PF, Song HF, Zhang Q, Yan CX. Pan-cancer immunogenomic analyses reveal sex disparity in the efficacy of cancer immunotherapy. Eur J Cancer. 2020; 126:136–38. 10.1016/j.ejca.2019.12.008 31927214

[11] Wang S, Zhang J, He Z, Wu K, Liu XS. The predictive power of tumor mutational burden in lung cancer immunotherapy response is influenced by patients’ sex. Int J Cancer. 2019; 145:2840–49. 10.1002/ijc.32327 30972745

[12] Weinrich SL, Pruzan R, Ma L, Ouellette M, Tesmer VM, Holt SE, Bodnar AG, Lichtsteiner S, Kim NW, Trager JB, Taylor RD, Carlos R, Andrews WH, et al. Reconstitution of human telomerase with the template RNA component hTR and the catalytic protein subunit hTRT. Nat Genet. 1997; 17:498–502. 10.1038/ng1297-498 9398860

[13] Lorbeer FK, Hockemeyer D. TERT promoter mutations and telomeres during tumorigenesis. Curr Opin Genet Dev. 2020; 60:56–62. 10.1016/j.gde.2020.02.001 32163830

[14] Osella-Abate S, Bertero L, Senetta R, Mariani S, Lisa F, Coppola V, Metovic J, Pasini B, Puig SS, Fierro MT, Manrique-Silva E, Kumar R, Nagore E, et al. TERT promoter mutations are associated with visceral spreading in melanoma of the trunk. Cancers (Basel). 2019; 11:452. 10.3390/cancers11040452 30934988 PMC6520836

[15] Simon M, Hosen I, Gousias K, Rachakonda S, Heidenreich B, Gessi M, Schramm J, Hemminki K, Waha A, Kumar R. TERT promoter mutations: a novel independent prognostic factor in primary glioblastomas. Neuro Oncol. 2015; 17:45–52. 10.1093/neuonc/nou158 25140036 PMC4483052

[16] Melo M, da Rocha AG, Vinagre J, Batista R, Peixoto J, Tavares C, Celestino R, Almeida A, Salgado C, Eloy C, Castro P, Prazeres H, Lima J, et al. TERT promoter mutations are a major indicator of poor outcome in differentiated thyroid carcinomas. J Clin Endocrinol Metab. 2014; 99:E754–65. 10.1210/jc.2013-3734 24476079 PMC4191548

[17] Leão R, Lee D, Figueiredo A, Hermanns T, Wild P, Komosa M, Lau I, Mistry M, Nunes NM, Price AJ, Zhang C, Lipman T, Poyet C, et al. Combined genetic and epigenetic alterations of the TERT promoter affect clinical and biological behavior of bladder cancer. Int J Cancer. 2019; 144:1676–84. 10.1002/ijc.31935 30350309 PMC6519346

[18] Batista R, Lima L, Vinagre J, Pinto V, Lyra J, Máximo V, Santos L, Soares P. TERT promoter mutation as a potential predictive biomarker in BCG-treated bladder cancer patients. Int J Mol Sci. 2020; 21:947. 10.3390/ijms21030947 32023888 PMC7037401

[19] Shin JY, Jung HJ, Moon A. Molecular markers in sex differences in cancer. Toxicol Res. 2019; 35:331–41. 10.5487/TR.2019.35.4.331 31636844 PMC6791665

[20] van Nas A, Guhathakurta D, Wang SS, Yehya N, Horvath S, Zhang B, Ingram-Drake L, Chaudhuri G, Schadt EE, Drake TA, Arnold AP, Lusis AJ. Elucidating the role of gonadal hormones in sexually dimorphic gene coexpression networks. Endocrinology. 2009; 150:1235–49. 10.1210/en.2008-0563 18974276 PMC2654741

[21] Vinagre J, Almeida A, Pópulo H, Batista R, Lyra J, Pinto V, Coelho R, Celestino R, Prazeres H, Lima L, Melo M, da Rocha AG, Preto A, et al. Frequency of TERT promoter mutations in human cancers. Nat Commun. 2013; 4:2185. 10.1038/ncomms3185 23887589

[22] Liu X, Wu G, Shan Y, Hartmann C, von Deimling A, Xing M. Highly prevalent TERT promoter mutations in bladder cancer and glioblastoma. Cell Cycle. 2013; 12:1637–38. 10.4161/cc.24662 23603989 PMC3680543

[23] Rachakonda PS, Hosen I, de Verdier PJ, Fallah M, Heidenreich B, Ryk C, Wiklund NP, Steineck G, Schadendorf D, Hemminki K, Kumar R. TERT promoter mutations in bladder cancer affect patient survival and disease recurrence through modification by a common polymorphism. Proc Natl Acad Sci USA. 2013; 110:17426–31. 10.1073/pnas.1310522110 24101484 PMC3808633

[24] Killela PJ, Reitman ZJ, Jiao Y, Bettegowda C, Agrawal N, Diaz LA Jr, Friedman AH, Friedman H, Gallia GL, Giovanella BC, Grollman AP, He TC, He Y, et al. TERT promoter mutations occur frequently in gliomas and a subset of tumors derived from cells with low rates of self-renewal. Proc Natl Acad Sci USA. 2013; 110:6021–26. 10.1073/pnas.1303607110 23530248 PMC3625331

[25] Bell RJ, Rube HT, Xavier-Magalhães A, Costa BM, Mancini A, Song JS, Costello JF. Understanding TERT promoter mutations: a common path to immortality. Mol Cancer Res. 2016; 14:315–23. 10.1158/1541-7786.MCR-16-0003 26941407 PMC4852159

[26] Lee HW, Park TI, Jang SY, Park SY, Park WJ, Jung SJ, Lee JH. Clinicopathological characteristics of TERT promoter mutation and telomere length in hepatocellular carcinoma. Medicine (Baltimore). 2017; 96:e5766. 10.1097/MD.0000000000005766 28151853 PMC5293416

[27] Egberts F, Krüger S, Behrens HM, Bergner I, Papaspyrou G, Werner JA, Alkatout I, Haag J, Hauschild A, Röcken C. Melanomas of unknown primary frequently harbor TERT-promoter mutations. Melanoma Res. 2014; 24:131–36. 10.1097/CMR.0000000000000048 24463461

[28] Conforti F, Pala L, Bagnardi V, Viale G, De Pas T, Pagan E, Pennacchioli E, Cocorocchio E, Ferrucci PF, De Marinis F, Gelber RD, Goldhirsch A. Sex-based heterogeneity in response to lung cancer immunotherapy: a systematic review and meta-analysis. J Natl Cancer Inst. 2019; 111:772–81. 10.1093/jnci/djz094 31106827 PMC6695312

[29] Wang S, Cowley LA, Liu XS. Sex differences in cancer immunotherapy efficacy, biomarkers, and therapeutic strategy. Molecules. 2019; 24:3214. 10.3390/molecules24183214 31487832 PMC6767080

[30] Wu Y, Ju Q, Jia K, Yu J, Shi H, Wu H, Jiang M. Correlation between sex and efficacy of immune checkpoint inhibitors (PD-1 and CTLA-4 inhibitors). Int J Cancer. 2018; 143:45–51. 10.1002/ijc.31301 29424425

[31] Grassadonia A, Sperduti I, Vici P, Iezzi L, Brocco D, Gamucci T, Pizzuti L, Maugeri-Saccà M, Marchetti P, Cognetti G, De Tursi M, Natoli C, Barba M, Tinari N. Effect of gender on the outcome of patients receiving immune checkpoint inhibitors for advanced cancer: a systematic review and meta-analysis of phase III randomized clinical trials. J Clin Med. 2018; 7:542. 10.3390/jcm7120542 30545122 PMC6306894

[32] Gao J, Aksoy BA, Dogrusoz U, Dresdner G, Gross B, Sumer SO, Sun Y, Jacobsen A, Sinha R, Larsson E, Cerami E, Sander C, Schultz N. Integrative analysis of complex cancer genomics and clinical profiles using the cBioPortal. Sci Signal. 2013; 6:pl1. 10.1126/scisignal.2004088 23550210 PMC4160307

[33] Zehir A, Benayed R, Shah RH, Syed A, Middha S, Kim HR, Srinivasan P, Gao J, Chakravarty D, Devlin SM, Hellmann MD, Barron DA, Schram AM, et al. Mutational landscape of metastatic cancer revealed from prospective clinical sequencing of 10,000 patients. Nat Med. 2017; 23:703–13. 10.1038/nm.4333 28481359 PMC5461196

[34] Samstein RM, Lee CH, Shoushtari AN, Hellmann MD, Shen R, Janjigian YY, Barron DA, Zehir A, Jordan EJ, Omuro A, Kaley TJ, Kendall SM, Motzer RJ, et al. Tumor mutational load predicts survival after immunotherapy across multiple cancer types. Nat Genet. 2019; 51:202–06. 10.1038/s41588-018-0312-8 30643254 PMC6365097

